# Treatment of Deciduous Teeth

**Published:** 1882-12

**Authors:** F. D. Wilson


### Treatment of the Deciduous Teeth.

DR. F. D. WILSON

[Transactions Michigan State Dental Society.]

The first and greatest difficulty we encounter, perhaps, is in securing the little patient upon which to practice the skill we may possess. Often the first appearance of the infant (?) in the office of the dental practitioner is for the purpose of leaving with him a carious temporary molar, as they think, but which proves to be one of the first members of the permanent set. The dentist who is the possessor of a number of children may consider himself favored in regard to this matter, at least for the experience it affords him.

Parents should be instructed to have their children's teeth examined at intervals, from the period that they first begin to erupt. A mere mention to them is not sufficient. Impress its importance upon their minds.

Mothers have a tenderness for their offspring, for which reason it is easy to find an excuse for neglect.

When a child is taken to a dentist, or he is called upon to visit it, he should first inspire it with confidence in himself. He should be kind, considerate, but firm. He should have a strict regard for the *truth*, and in *no* particular deceive a child.

It should not only be discouraged, but absolutely forbidden, for the parent to do so. Tell the child what you intend to do, as near as possible, that it may be painful, and encourage it to bear it. To lose its confidence in you is as bad as to lose the patient.

Let us now examine the jaws of a fœtus. The process of formation of the teeth previous to the deposition of lime is divided into three stages. From six to ten weeks is the papillary stage; from ten to fourteen the follicular; and from fourteen to fifteen the saccular. From the sixteenth to the twenty-first week the deposit of lime begins. The teeth are first gradually formed in soft tissues, the crowns becoming calcified before the roots are entirely formed in soft tissue. At birth the two superior maxillar are united by a fibro cartilage.

At the period of the eruption of the first teeth, the crowns of the central incisors are perfected and the lateral incisors are almost equally advanced. The crowns of the cuspids are, as yet, incomplete. The crowns of the first molars are near their completion, but the second molar is somewhat less advanced than the cuspids.

The process of the growth and absorption of the alveoli is interesting. It is first built up to enclose the teeth in process of formation. They erupting, it is absorbed to admit of the passage of their crowns. It is now rebuilt to firmly secure the teeth in position. In the eruption of the permanent set it is again absorbed and rebuilt to form their sockets.

The process of dentition is a normal one, and should take place without any unpleasant or dangerous symptoms; but there are few children who pass through this period without suffering in a greater or less degree.

The teeth vary a great deal in their time of eruption as regards different individuals. A premature eruption of them is much more liable to result in some disturbance than if their eruption is retarded.

The teeth erupt in groups. The first group appears about seven months after birth, and consists of the two inferior central incisors.

Next come the four superior incisors at nine or ten months, and constitutes the second group.

Now comes quite a long interval.

The next teeth to erupt are the two inferior lateral incisors and the first four molars, at from twelve to fifteen months. They are the third group.

The fourth group consists of the four cuspids, which appear from the eighteenth to the twenty-fourth month.

The fifth and last group includes the remaining teeth, the four second molars, and erupt from thirty to thirty-six months.

It has been a matter of some dispute as to whether the cuspids or the first molars are the cause of the most disturbance. The cuspids are the only ones that come in between two others. Mortality tables show that one out of every five infants dies before reaching the age of twelve months; and one out of every three children dies during the first five years of its life.

Now, out of this number, it has been asserted that over four per cent. of those dying during the first year, that death was caused from diseases or affections resulting from dental irritation, and that seven per cent. of the deaths occurring between the ages of one and three years were from the same cause. It is thought, however, by later writers and practitioners that this is much exaggerated.

The causes of a deranged dentition are numerous.

The general health of the infant plays a very important part.

Physicians, as a rule, do not give the study and attention to the diseases of children that the subject demands, while a large proportion of their practice is among them.

The great predisposing cause of a disordered dentition is of course a lowered vitality of the system. Feeble children are more likely to suffer than robust ones. Living in crowded cities where the atmosphere is always more or less vitiated, food insufficient in quantity or of inferior quality, must not be overlooked as predisposing causes, and it is very necessary that they should be corrected. Cleanliness is also of great importance. It is very necessary that the child be properly clothed. On the other hand, a child may be overfed, and experience evil results from that cause.

Too particular care should not be taken of children. A new born babe may be taken out in the air for several hours a day if the weather admits of it.

There seems to be no doubt that the process of dentition is charged with more mischief than is due to it. There are various affections which may be, and often are due to it; but may all be produced from some other cause.

In the infant the nervous system predominates. The salivary glands are comparatively inactive and do not secrete saliva proper. Perfect saliva is secreted at about three months. The child cannot digest starchy food until this period. The alimentary canal is comparatively shorter in the child. It is best to wean a child between the period of eruption of the second and third groups of teeth—ten to twelve months. It may begin to take solid food when teething commences, and when weaned let it be done somewhat gradually.

The secretions of the skin in infancy are extremely active; and from a lack of control of the sebaceous secretions, we have the occurrence of many skin diseases.

An erysipelatous mother should not nurse her child. A syphilitic mother may.

In the infant the stomach is small and straight, it is simply a dilation of the alimentary canal. It is not suited to retain food long, and the digestion is quick. The developement of the alimentary canal keeps pace with the teeth.

Rachitis, or more familiarly richets, is a condition of the system that much interferes with dentition. Its treatment is mostly hygienic, tonics probably are indicated.

Tubercule does not interfere with dentition. The alveolar ridges of the jaws may be small and unusually porous. There may be a lack of the proper amount of process, while it may still be normally compact. In these cases the anterior surfaces of the teeth can be felt through the gums. In the latter case dentition is easy- The irritation of an erupting tooth may disappear for a time and then return. This, perhaps, is caused by the resistance of the process first, and then the resistance of the soft parts. The cases are rare where there is irritation from advancing teeth, and no local indications of it exist.

The dental difficulty itself may be but a symptom. The irrita tion from dentition may cause:

1. A stomatitis.
2. Irruptive fevers.
3. Spasms.
4. Eruptions of the skin—especially of the scalp and face.

In the first of these (stomatitis) the inflammation may be a localized or diffused one, depending upon the degree of irritation, and the susceptibility of the child to such impression.

The first symptoms are an uneasy, itching sensation of the gums, an increased flow of saliva, and tumefaction of the gums.

Now comes the question as to whether the lancet shall be used. If the child seems otherwise healthy, the swelling over the advancing tooth presents a glistening tense appearance, it is advisable to use the lancet in cases of the incisor teeth cutting in the direction of the arch, in the molars, making a crucial incision. A propei position for the patient and operator to be in, is to have the child sit upon the nurses lap with its face toward hers; the operator sitting facing the nurse, draws the head and body of the child backwards, and has then good control of it.

The gum lancet, though often serving an excellent purpose, is an instrument not nearly so much in demand as in the past Not only is its use not indicated, but it often aggravates the difficulty it is intended to relieve. Palliative treatment is preferable. Tincture of Belladonna, or a saturated solution of Potassium Bromide, applied to the gums will often suffice.

A little sack of ice held in the mouth will frequently give much relief. When the lance is used a cicatrix may form over the tooth which is more resistant than before the cutting. Hemorrhage is seldom troublesome after the proper use of the lance. If the bleeding is too profuse mild astringents may be used, and internal medicament also

The sucking propensity of infants is to be guarded against by the insertion of some article in the mouth to prevent its being able to form a vacuum. The dental irritation may be but the exciting cause of the disturbance, and only a small part of the treatment will therefore be directed to the teeth. Inflammation of the mouth may extend by continuity to the various parts lined by mucous membrane, or the irritation may excite a morbid action in almost any organ in the body.

The febrile state is perhaps the most frequent results of a disordered dentition. There is usually more or less fever. It is remarkable for the sudden rise and declination often. It is attended with much restlessness. The sleep is broken, thirst is great, and the appetite impaired. Our diagnosis will sometimes be difficult, we must look for causes aside from the teeth. Our treatment will be governed by the indications presented, though the same general treatment will be applicable to a majority of the cases. Refrigerating drinks are often very soothing and beneficial. Emetics and cathartics may be used with good effect. Potassium Bromide is often invaluable. If our treatment be not successful, we must look to discover if there are any surrounding or outside influences.

Diarrhœa so frequently occurring in the infant, is perhaps not as often the result of dental irritation as is generally supposed. The mucous membrane of the mouth, being continuous with the œsophagus, stomach and intestines, it will be seen that an inflammation at one point may cause the whole tract to become irritable. Food in excess, or of improper quality, worms, Enteritis. Tabes Mesenterica are among the causes of diarrhœa. Diarrhœa occurs more frequently in hot weather. The milk of a nurse is sometimes the cause of diarrhœa. The nursing bottle is often in an unwholesome condition. After discovering, if possible, and removing the cause, medicines will often hasten the restoration to the normal condition. A long list of medicines have been resorted to for the relief of diarrhœa. Opium is perhaps one of the best. The administration of sweet oil and paregoric is a good form for the child; or small doses of Dover's powder. The citrate and tartrate of Potassium are also serviceable. Warm applications to the abdomen are often of good service. If the diarrhœa is not inflammatory in character the Bromide is indicated.

Spasms should here receive some notice. It is considered a direct or indirect irritation of the spinal cord or its branches. Here, perhaps, a little more liberty in lancing the gums will be allowed. We may cut down upon teeth whose period of eruption is at hand. It is good practice to insert into the incision a pledget of cotton saturated with a solution of sulphate of morphia. If the child is anæmic, tonics are indicated in connection with sedatives. If the child is plethoric, Potassium Bromide and the salines are indicated.

All stimulating food should be avoided. Hot foot baths will often be of service. The abstracting of blood by leeches, or opening a vein may be necessary.

Spasm usually requires speedy relief. A babe in convulsion may be immersed in a warm bath of 75 degrees. Emetics which are not depressing should be used to rid the stomach of any undigested food. Chloral Hydrate is of great value, and if necessary chloroform may be given to the babe.

I am unable to say to what extent the irritation of teething affects the skin. It is accountable for a share of such affections. Its consideration would make a long paper of itself. Dental irritation does not produce any skin disease peculiar to that cause.

